# Standard EF tasks can still have predictive validity within diverse cultural contexts

**DOI:** 10.1073/pnas.2536826123

**Published:** 2026-04-06

**Authors:** Jesse C. Niebaum

**Affiliations:** ^a^Center for Mind and Brain, Department of Psychology, Davis, CA 95616

Kroupin et al. (1) administered standard executive function (EF) tasks to children in the United Kingdom, Bolivian children attending “limited” schooling, and Kunene children in Namibia attending or not attending school. Children in the United Kingdom performed best on these assessments, whereas unschooled Kunene children performed worst, often never providing expected responses. The authors conclude that standard EF tasks, created and normed in industrialized and schooled countries, capture cognitive skills that develop in those contexts (see also refs. 2–5 for discussion). I agree with Kroupin et al. that unmeasured cultural factors likely drive the observed mean differences in EF task performance; different cultural affordances lead to specialized forms of EF engagement, which in turn influence scores on standard EF assessments across diverse samples (6–8). However, additional analysis of Kroupin et al. data indicates that standard EF tasks capture theoretically and practically meaningful variation in children’s EF, even in these less industrialized samples.

Kroupin et al. (1) dataset includes measures of reading and math for the Bolivian and Kunene children attending school, in addition to age and years of schooling. Additional analysis of these data reveals two key patterns beyond those described in the article. First, EF task performance increased with age in the Bolivian and both Kunene samples, across EF assessments; age remained a significant predictor of performance for many EF assessments in the Bolivian and schooled Kunene samples even when controlling for years of schooling (Table 1). Second, EF task performance correlated with math and reading scores in each of the schooled samples; many of these correlations persisted even when controlling for years of schooling (and 17/18 coefficients remained positive; Table 2). If EF task performance primarily reflects school exposure, then these correlations should disappear when controlling for years in school. Instead, age-related increases were observed in all samples, and correlations with academic scores remained in the schooled samples, consistent with the interpretation that standard EF tasks capture functionally meaningful aspects of EF engagement and not merely school exposure or development in more industrialized cultures.

**Table 1.** Associations between cognitive task performance, age, and years in school

		Simple age effects	Age effects controlling for years in school	
EF Task	Group	Age	Age	Years in School
Forward Span	Schooled Kunene	**0.06 (0.03)** ^*^	0.05 (0.04)	0.02 (0.05)
	Bolivian	**0.06 (0.02)** ^*^	0.02 (0.05)	0.05 (0.06)
	Unschool Kunene	**0.10 (0.03)** ^*^		
Backward span	Schooled Kunene	**0.13 (0.03)** ^*^	**0.15 (0.04)** ^*^	–0.04 (0.06)
	Bolivian	**0.20 (0.05)** ^*^	0.04 (0.10)	0.23 (0.12)
	Unschool Kunene	**0.21 (0.04)** ^*^		
Dimensional change card sort^†^	Schooled Kunene	–	–	–
	Bolivian	**0.07 (0.02)** ^*^	**0.08 (0.04)** ^*^	−0.02 (0.05)
	Unschool Kunene	**0.06 (0.02)** ^*^		
Luria’s hand test trials	Schooled Kunene	**0.32 (0.15)** ^*^	0.03 (0.19)	**0.61 (0.27)** ^*^
	Bolivian	**0.31 (0.00)** ^*^	0.27 (0.22)	0.05 (0.26)
	Unschool Kunene	**0.51 (0.20)** ^*^		
Verbal fluency	Schooled Kunene	**0.83 (0.11)** ^*^	**0.52 (0.13)** ^*^	**0.64 (0.19)** ^*^
	Bolivian	**0.61 (0.13)** ^*^	**0.68 (0.29)** ^*^	−0.09 (0.35)
	Unschool Kunene	**0.92 (0.12)** ^*^		
Imitation Task				
Luria’s hand imitation trials	Schooled Kunene	0.07 (0.70)	0.08 (0.09)	−0.02 (0.13)
	Bolivian	0.05 (0.05)	0.01 (0.10)	0.05 (0.13)
	Unschool Kunene	0.11 (0.09)		

^*^P < 0.05.

^†^Effects estimated with a generalized logistic regression because outcomes were coded as 1 or 0. Effects for Schooled Kunene children not included because 91% scored 1.

**Table 2.** Association between cognitive task performance and academic performance

EF Task	Group	Math	Math (Controlling for years in school)	Reading	Reading (Controlling for years in school)
Forward span	Schooled Kunene	**0.60 (0.29)** ^*^	**0.59 (0.22)** ^*^	**0.72 (0.31)** ^*^	0.26 (0.24)
	Bolivian	**0.79 (0.26)** ^*^	0.36 (0.23)	**1.15 (0.33)** ^*^	**0.60 (0.29)** ^*^
Backward span	Schooled Kunene	**0.84 (0.23)** ^*^	**0.50 (0.18)** ^*^	**0.71 (0.24)** ^*^	0.38 (0.19)
	Bolivian	**0.36 (0.11)** ^*^	0.05 (0.11)	**0.74 (0.14)** ^*^	**0.41 (0.14)** ^*^
Dimensional change card sort^†^	Schooled Kunene	–	–	–	–
	Bolivian	**0.82 (0.38)** ^*^	0.13 (0.33)	**1.01 (0.48)** ^*^	0.11 (0.41)
Luria’s hand test	Schooled Kunene	**0.17 (0.05)** ^*^	0.06 (0.04)	**0.21 (0.05)** ^*^	**0.10 (0.04)** ^*^
	Bolivian	**0.14 (0.06)** ^*^	0.03 (0.05)	0.01 (0.08)	-0.05 (0.07)
Verbal fluency	Schooled Kunene	**0.29 (0.05)** ^*^	**0.13 (0.05)** ^*^	**0.27 (0.05)** ^*^	0.10 (0.06)
	Bolivian	**0.11 (0.04)** ^*^	0.00 (0.39)	**0.13 (0.06)** ^*^	0.00 (0.05)
Non-EF Task					
Luria‘s imitation test	Schooled Kunene	−0.02 (0.12)	−0.07 (0.09)	0.07 (0.16)	0.02 (0.09)
	Bolivian	0.07 (0.14)	−0.02 (0.11)	0.16 (0.18)	0.04 (0.14)

^*^P < 0.05.

^†^Effects for Schooled Kunene children not included because 91% scored 1.

It is possible that factors associated with attending school beyond years of attendance drive both EF task performance and academic scores, and different aspects of EF task performance may underlie the predictive validity of EF task performance across diverse cultures. However, such explanations must account for why *individual differences* in EF task performance predict *individual differences* in outcomes like academic performance. Further cross-cultural work on EF development that incorporates other culturally relevant outcomes is still needed to determine whether the predictive validity of EF assessments extends beyond academic contexts, as it does in Western samples (9). Regardless, the analysis presented here indicates that differences in mean EF task performance across cultural groups do not preclude standard EF tasks from capturing meaningful variation in how children engage EF *within* cultural groups. Combined with the results reported in Kroupin et al., these findings support a conceptualization of EF and its measurement as *both* universal and culturally specific (10).
